# Postaxial Polydactyly Type B in Infants: A Narrative Review of Outcomes and Family-Centered Care

**DOI:** 10.7759/cureus.117353

**Published:** 2026-10-02

**Authors:** Stephen J Gauta, Bryan Bartnick, Luis P Luy, Brett Shannon, Jordan Vokes

**Affiliations:** 1 Medical Education, Albany Medical College, Albany, USA; 2 Pediatric Orthopaedics, Lee Memorial Health System, Fort Myers, USA

**Keywords:** caregiver satisfaction, congenital hand anomaly, local anesthesia, postaxial polydactyly, shared decision-making, surgical excision, suture ligation, ulnar polydactyly

## Abstract

Postaxial polydactyly type B is a rudimentary ulnar-sided digit attached by a soft-tissue pedicle. Although treatment usually preserves hand function, families must weigh differences in timing, anesthesia, residual appearance, symptoms, and the possibility of another procedure. This narrative review examines the evidence for suture or clip ligation and surgical excision, with particular attention to caregiver experience and the limits of psychosocial outcome data. Targeted literature searches and reference-list review were updated in September 2026. Systematic reviews and observational cohorts generally associate excision with fewer composite complications and less residual prominence, while satisfaction is high after several treatment approaches. Early excision under local anesthesia offers immediate removal and direct management of the neurovascular pedicle when appropriate expertise and facilities are available. Ligation remains an option for selected narrow attachments after discussion of delayed separation and possible residual tissue or revision. Caregiver studies support discussing treatment timing, anesthesia concerns, and satisfaction with appearance; they do not establish that excision prevents childhood bullying, anxiety, or depression. A visible remnant, caregiver dissatisfaction, and child distress should therefore be assessed separately. An anatomy illustration and an author-developed treatment decision aid connect these distinctions to clinical assessment, shared decisions, and follow-up. The pathway includes observation or planned excision when immediate treatment is deferred. Better prospective studies should distinguish initial treatment from revision and measure caregiver concerns separately from the child's later experience.

## Introduction and background

Postaxial polydactyly type B is a rudimentary extra digit on the ulnar side of the hand. Its soft-tissue pedicle contains small vessels and an accompanying sensory nerve, and the condition is often bilateral and familial [1,2]. Most cases are isolated, although reported frequency varies among populations [1,2]. Postaxial polydactyly type B differs from postaxial polydactyly type A, which has a developed skeletal connection and requires a different operative assessment [3]. Recognizing this distinction determines whether a simple removal procedure is appropriate.

There are two main methods of removal: suture or clip ligation and surgical excision. Practice varies among clinicians and treatment settings, although contemporary pediatric hand-surgeon survey data favor early office excision [4]. Ligation is familiar and readily available, while excision permits immediate removal and direct management of the neurovascular pedicle. Systematic reviews generally favor excision for composite complications, but the underlying studies are heterogeneous and predominantly observational [5,6]. Families may value rapid treatment, avoidance of general anesthesia, or the outcome experienced by an affected relative. These priorities deserve discussion alongside the clinical evidence.

This narrative review brings together treatment outcomes, caregiver perspectives, and practical considerations for infant type B disease. We focus on a distinction that matters during counseling: a residual bump, pain, dissatisfaction with appearance, and psychological distress are different outcomes. We also present an educational treatment decision aid that links anatomic assessment to treatment selection and follow-up. The aim is to help clinicians explain the available choices without attributing unmeasured benefits to either procedure.

## Review

Approach to the literature

The review used targeted searches of PubMed/MEDLINE, Google Scholar, and reference lists for postaxial or ulnar polydactyly, type B, ligation, excision, local anesthesia, complications, satisfaction, and treatment setting. The original literature search was updated through July 18, 2026. Focused searches of Europe PMC, which includes PubMed-indexed records, and publisher sources were performed on September 10-11, 2026, to update treatment evidence and reassess caregiver and psychosocial outcomes. Additional terms included parent, caregiver, appearance, distress, anxiety, psychosocial, and bullying. Citation details and key findings were checked against indexed records and available full texts.

Priority was given to systematic reviews, comparative cohorts, longitudinal studies, caregiver surveys, and clinically relevant case series or technique reports. The treatment synthesis concerns hand type B disease; evidence from other hand differences or foot polydactyly was not used to estimate its psychological burden. Two authors collaborated in identifying and discussing the literature during the original review. Screening and extraction were not independently duplicated, no protocol was registered, and no formal risk-of-bias instrument was applied. This is a narrative synthesis, with pooled estimates attributed to the published reviews rather than calculated anew.

Epidemiology and initial assessment

The reported frequency of type B polydactyly varies across populations. In a cohort of 11,161 newborns, 21 infants were affected; 76% had bilateral involvement, and 86% had a family history [2]. A larger surveillance study identified 545 affected infants, 95% of whom had an isolated anomaly. Reported frequencies in that population were 0.91 per 100 Black infants and 0.035 per 100 White infants [1]. These estimates describe the populations studied and should not be used to determine the treatment offered to an individual infant.

The initial examination should document laterality, attachment width, skin integrity, perfusion, family history, and any previous trauma or attempted treatment. Both hands and feet and the infant's general examination should be assessed. A broad base, palpable bone or joint, meaningful movement, or uncertain anatomy should prompt specialist assessment rather than routine ligation [1,3]. Radiographs are useful when a skeletal connection is suspected; an unequivocal narrow soft-tissue attachment can usually be assessed clinically. The importance of morphology is supported by a multicenter registry of 334 patients with ulnar polydactyly, in which an associated syndrome was reported in 2% of type B cases and 21% of type A cases [7]. Because these were referral-based observations, they should inform careful assessment rather than population-level risk prediction. Associated anomalies or atypical findings warrant pediatric, genetic, or hand-surgery evaluation as appropriate. The neurovascular anatomy illustrated in Figure 1 explains why even a small, nonfunctional appendage requires attention to both bleeding control and the sensory nerve.

**Figure 1:**
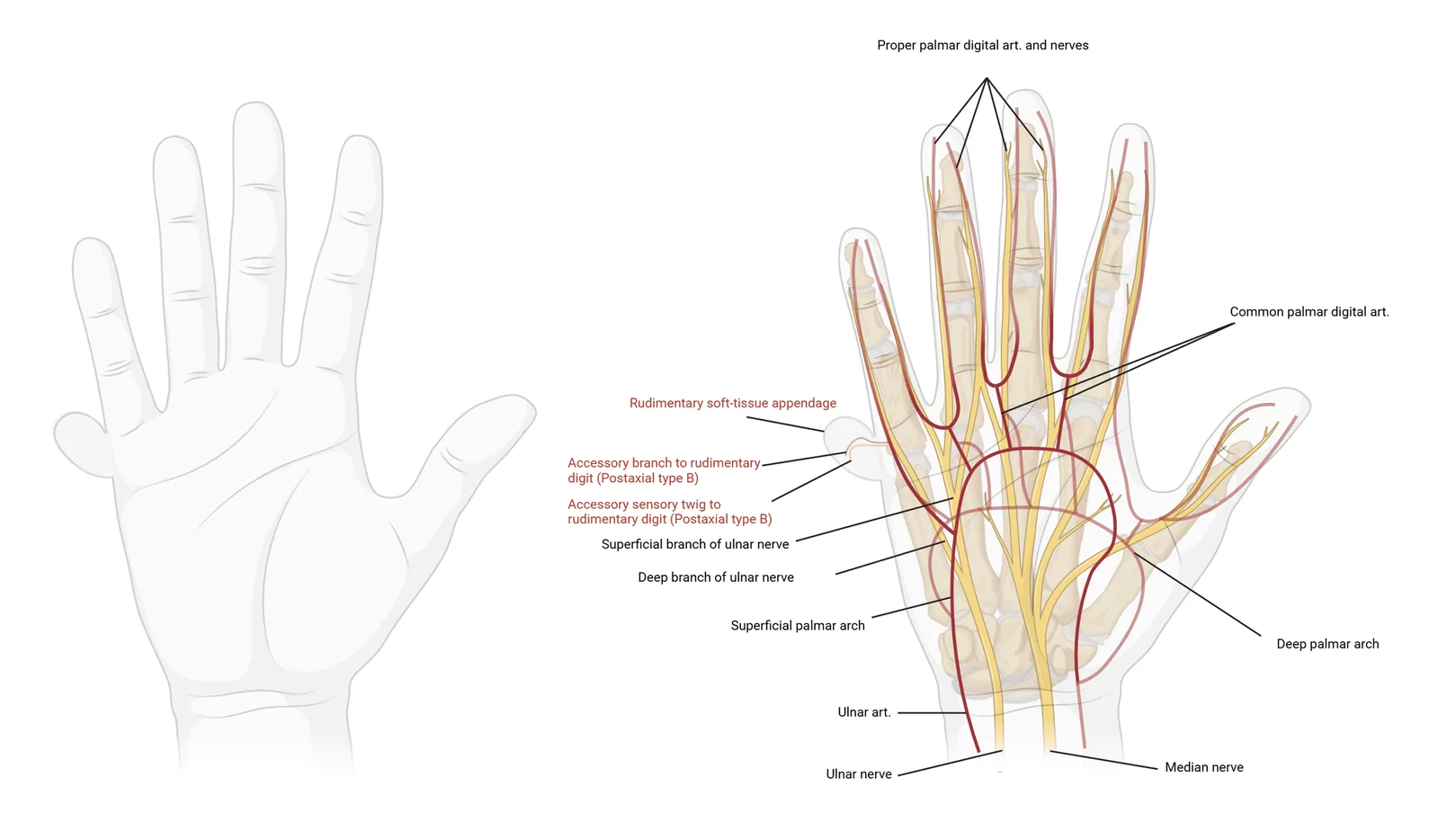
Anatomy and presentation of postaxial polydactyly type B The left panel shows a rudimentary ulnar-sided appendage; the right panel illustrates the accompanying neurovascular connection. The accessory digit lacks a skeletal connection. The sensory nerve is relevant to the assessment of a tender remnant [1,3]. Image Credit: Hand Illustration. Created manually by Stephen J. Gauta (2026) using BioRender (Toronto, Ontario, Canada) (https://BioRender.com/guhcxfy)

Suture and clip ligation

Ligation occludes the pedicle and allows ischemic autoamputation over the following days. Its advantages are immediate availability, limited equipment, and avoidance of an incision. It is anatomically suitable only for a very narrow soft-tissue attachment. Treatment starts immediately, but removal is delayed until separation occurs. Incomplete separation and residual prominence are recognized sequelae [2,5,6]. Counseling should explain this sequence and provide instructions about wound care, bleeding, infection, and access to reassessment.

Suture and clip techniques should be discussed separately when the evidence permits. A retrospective clip series followed 132 newborns with 231 treated hands for at least two years; scar revision was performed in 16 extremities among nine patients [8]. Reporting both denominators matters because patient-level and digit-level revision rates are not interchangeable. Symptomatic traumatic amputation neuromas have also been described after suture ligation, consistent with an accessory nerve ending remaining within a superficial scar [9]. These reports establish that painful remnants can occur, but they do not provide a universal neuroma rate. A visible bump alone should not be labeled a neuroma.

Excision under local anesthesia

Excision removes the digit immediately, permits direct hemostasis, and allows the accessory sensory nerve to be managed proximal to the skin closure. Nursery or office treatment can avoid general anesthesia in selected infants. Feeding and swaddling may support a local-anesthetic protocol, but do not replace procedural analgesia [10]. Age limits, anesthetic dosing, monitoring, and preparation should follow the treating service's protocol. Appropriate experience, assistance, lighting, and the ability to control bleeding are prerequisites.

Nursery and office series have reported favorable healing and cosmetic outcomes [11,12]. In one office cohort, 78 digits were excised in 48 children without intraoperative or early postoperative complications; parents also reported favorable outcomes at a mean telephone follow-up of 3.2 years [13]. A separate comparison of local and general anesthesia in 91 infants found lower costs and shorter recovery-related time measures with local anesthesia [14]. Together, these findings support the feasibility of local-anesthetic excision in experienced hands. They do not establish a single age cutoff or make office excision appropriate for every infant.

Treatment setting and access

Operating-room excision remains appropriate when anatomy, infant tolerance, a concurrent operation, or the local service's capabilities make office treatment unsuitable. Anesthesia and location are separate decisions: local anesthesia can also be used in an operating room [6,14]. Families should understand the actual proposed anesthetic, preparation, expected wait, and recovery, rather than assuming that all surgical excision requires general anesthesia.

Local-anesthetic treatment may reduce delays as well as the resources required for the procedure. A retrospective study of 55 children reported shorter waits and procedure-related time measures with local anesthesia [15]. In a larger analysis of 620 patients, including 590 office and 30 operating-room excisions, office treatment was associated with lower charges and insurer payments. Recorded complications and patient payments did not differ significantly, although substantial differences in patient age and size limited the clinical comparison [16]. Another cost analysis comparing 64 office and 50 operating-room procedures also found lower payer charges and health-system costs with office excision, but it did not measure clinical outcomes [17]. These findings support efficient service organization; they do not justify promising a particular family lower out-of-pocket costs.

Appointment availability, transport, insurance, and time away from work can affect the feasible choice. Clinicians can clarify referral timing and anticipated charges before a decision is made. These individual care considerations apply across settings. When timely office excision is unavailable, the discussion should include planned excision, selected ligation, or observation with reassessment.

Outcomes and sequelae

Hand function, residual contour, pain, revision, and satisfaction should be evaluated separately. A systematic review of 10 studies found substantial heterogeneity in outcome definitions and limited comparative evidence [5]. A subsequent review of 15 studies estimated pooled complication rates of 22% after ligation and approximately 1% after excision, corresponding to an odds ratio of 6.89 with a wide 95% confidence interval of 1.73-27 [6]. Although these pooled findings favor excision, differences in study quality, definitions, and follow-up prevent their use as precise estimates of an individual infant's risk.

A 2026 review of 14 studies comprising 885 patients and 1,279 digits found high parental satisfaction across treatments. Local-anesthetic excision had a statistical advantage, but the overall differences were small, and parent-reported outcomes were unavailable for vascular clips [18]. Because the abstract and main results report inconsistent pooled estimates for suture-ligation complications, we rely on the review's qualitative findings rather than reproduce a single percentage. Successive reviews also contain overlapping cohorts and should not be interpreted as independent replication in entirely new populations.

Residual tissue is not necessarily troublesome. In one follow-up examination, bumps were present in 12 of 28 fingers after ligation, yet all parents were satisfied with the appearance [2]. Conversely, a tender remnant can represent a symptomatic neuroma and may require further treatment [9]. Excision also creates a scar and cannot guarantee an imperceptible result. Describing appearance and symptoms separately is therefore more useful than treating every postoperative finding as a complication.

Long-term follow-up provides a more qualified picture. One study obtained responses from 69 of 173 eligible patients, a 40% response rate, at a mean of 11.1 years after treatment. Among the 24 respondents initially treated with ligation, 10 later underwent surgery for a symptomatic neuroma stump or persistent polydactyly. Treatment satisfaction favored excision, whereas hand-function scores and appearance ratings did not differ significantly [19]. The resulting 42% revision proportion applies only to this small responding cohort and should not be presented as the expected failure rate for all infants; response bias may have influenced the estimate.

A family survey with a 53% response rate captured outcomes for 258 digits in 158 children at a median age of eight years. Bumps or raised scars were reported in 50% of ligated digits and 34.4% of excised digits, while pain or sensitivity was reported in 12.1% and 11.5%, respectively [20]. Interpretation is limited because 18 digits initially treated with ligation and later excised were assigned to the excision group. The comparison therefore reflects final treatment rather than initial strategy. The survey also combined bumps with raised scars and did not clinically confirm neuromas. Similar reported pain deserves attention, but it does not establish equivalence between primary ligation and excision.

Taken together, the available evidence supports offering early local-anesthetic excision as a preferred definitive option when the infant and service are suitable. Its clearest advantages are immediate removal and lower rates of residual prominence or composite complications. The evidence is less certain for the prevention of long-term pain. Families should not be promised superior function, a scar-free result, or freedom from revision, and each of these outcomes should be discussed separately.

Caregiver experience and psychosocial considerations

Psychosocial counseling becomes more useful when caregiver concerns are distinguished from the child's later experience. Evidence from congenital hand differences indicates that parental aesthetic concerns may differ from a child's own priorities [21]. This principle is relevant to counseling, but the broader literature does not establish mental-health outcomes for infants with hand type B disease. Direct evidence for this condition is narrower and addresses treatment preferences, satisfaction with appearance, and the experience of waiting for or undergoing treatment.

Treatment choice has been examined prospectively in a small cohort of 14 newborns whose families were offered observation, suture ligation, immediate local-anesthetic excision, or delayed excision under general anesthesia. Most families chose immediate excision, and satisfaction was high across the treatments selected [22]. Earlier removal was proposed as a way to reduce parental distress or embarrassment, but the study was nonrandomized and had short follow-up. It therefore does not demonstrate a sustained psychological benefit. Its findings instead support asking families how the timing and experience of treatment affect them, rather than assuming that they will prefer the option that appears least invasive.

The experience of obtaining treatment also shapes family preferences. In a survey completed by 70 of 156 eligible families at a mean of 27 months after treatment, those who chose ligation more often identified rapid treatment as important. Residual tissue was associated with lower parental satisfaction with appearance and treatment, although most parents remained highly satisfied overall [23]. Generalizability is limited by the 45% response rate, retrospective recall, and the treatment choices available at that institution. Counseling should therefore address both the procedure and the practical experience of receiving it. A choice between immediate ligation and delayed operating-room excision may not reflect what the same family would choose if timely office excision were available.

The child's burden should be assessed directly when developmentally possible. In one family survey, more than half of respondents who reported a bump or scar said that it never bothered the child [20]. Appearance alone should therefore not be assumed to cause distress. We identified no comparative study showing that treatment of infant hand type B polydactyly prevents later bullying, anxiety, or depression. Evidence from broader congenital differences, persistent adolescent deformities, or foot polydactyly cannot establish that benefit for this population.

Clinically, these findings support a measured discussion of appearance without predicting harm or dismissing a family's concern. Clinicians should ask what worries the caregivers, explain what is known about scarring and symptoms, and avoid describing a visible difference as inevitably stigmatizing. An older child should be asked separately about discomfort, unwanted attention, and preferences regarding further treatment. Reported distress can be addressed on its merits, including supportive counseling when needed, but it should not automatically become an indication for revision. The available evidence informs caregiver counseling; it does not establish the prevention of bullying, anxiety, or depression.

Treatment decision aid and follow-up

Figure 2 presents an author-developed educational decision aid informed by the reviewed literature [5,6,18-20,22,23]. It begins with anatomic confirmation and routes broad-based, skeletal, or uncertain cases toward specialist assessment. For typical type B disease, clinicians should explain the procedures, offer a recommendation, and establish the family's priorities before selecting treatment. Early local excision is preferred when clinically suitable and chosen after discussion. Deferral does not automatically lead to ligation: a very narrow pedicle may permit ligation, while other circumstances may favor observation or planned excision. The pathway has not been prospectively validated.

**Figure 2:**
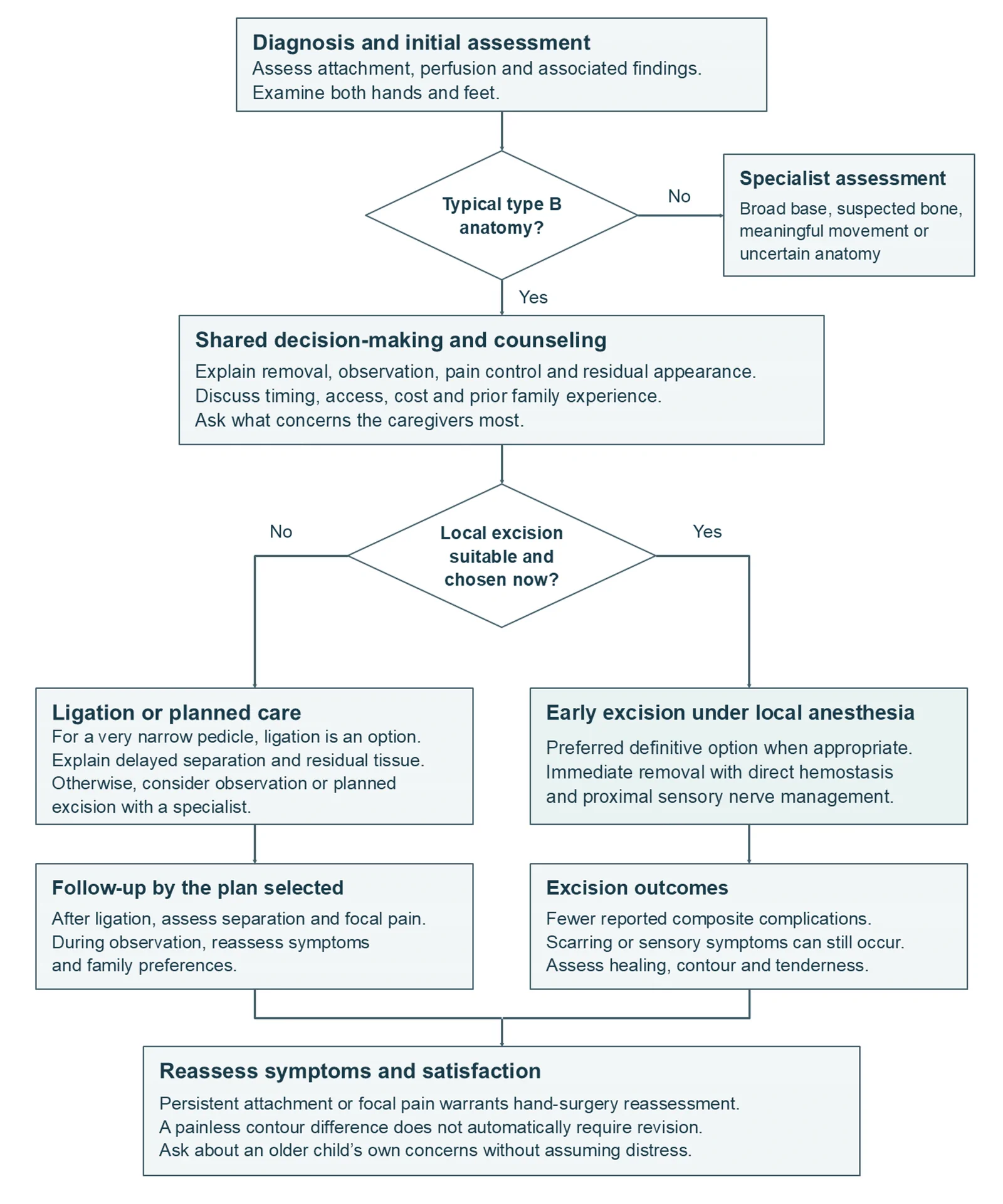
Treatment decision aid for infant postaxial polydactyly type B Author-developed educational synthesis of clinical and caregiver evidence [5,6,18-20,22,23]. A typical type B attachment is a narrow soft-tissue pedicle without a skeletal connection. Choice of treatment depends on clinical suitability, local expertise, availability, and informed family preferences. Deferral of local excision does not automatically indicate ligation. The pathway has not been prospectively validated. No treatment has been shown to prevent bullying or later psychological distress in this population.

Follow-up should confirm separation or wound healing after treatment and assess persistent attachment, bleeding, infection, focal tenderness, and satisfaction. Persistent symptoms warrant examination for residual tissue, scar sensitivity, or a possible neuroma [9,19,20]. An asymptomatic contour difference does not automatically require another procedure. When treatment is deferred, reassessment should address new symptoms and changing preferences. At either stage, caregiver satisfaction and an older child's own concerns should be recorded separately.

Limitations and future directions

This review is limited by targeted study selection, the absence of independent duplicate extraction, and the lack of a formal risk-of-bias assessment. The evidence is dominated by observational cohorts and case series. Anatomy, age, clinician preference, and service availability influence treatment selection, while inconsistent definitions and incomplete follow-up complicate comparisons [5,6,18-20]. Several reports use nonvalidated parent surveys; bilateral digit analyses can also overstate precision if correlation within a child is ignored. The decision aid organizes existing evidence and clinical reasoning without establishing a new standard of care.

Future studies should record the initial treatment and every subsequent intervention, distinguish painless residual tissue from symptomatic neuroma, and use consistent follow-up intervals. Family outcomes should include reasons for treatment choice, the experience of waiting and separation, satisfaction, and actual household costs. Child-reported symptoms, appearance concerns, and peer experiences should be collected separately when age-appropriate. Comparing these domains would clarify whether dissatisfaction reflects the clinical result, the process of care, or a concern experienced by the child. Such evidence would strengthen counseling more than a composite complication rate alone.

## Conclusions

Postaxial polydactyly type B requires accurate assessment of the attachment and a clear explanation of available treatments. Early excision under local anesthesia is a reasonable preferred definitive option when appropriate expertise and facilities are available. Ligation remains suitable for selected narrow pedicles after discussion of delayed separation, residual tissue, and possible further treatment. Observation or planned excision can be considered when immediate treatment is deferred.

Counseling should distinguish appearance, pain, function, caregiver satisfaction, and the child's own experience. Caregiver concerns about timing and appearance are legitimate, but current evidence does not show that excision prevents bullying or later psychological distress. A family-centered discussion can acknowledge these concerns while keeping treatment recommendations grounded in measured outcomes. Prospective studies that separate these domains are needed to make the benefits and limitations of each choice more precise.
